# Impact of Copper on the Physiology and Transcriptome of *Methylosinus Trichosporium* OB3b Grown on Either Methane or Methanol

**DOI:** 10.1111/1462-2920.70245

**Published:** 2026-01-16

**Authors:** Peng Peng, Jeremy D. Semrau

**Affiliations:** ^1^ Department of Civil and Environmental Engineering University of Michigan Ann Arbor Michigan USA

## Abstract

*Methylosinus trichosporium* OB3b is a model methanotroph that converts methane to CO_2_ with methanol, formaldehyde, and formate as intermediates. Here we show that copper has a significant impact on the ability of *Msn. trichosporium* OB3b to grow on methanol, that is, growth consistently occurred on methanol in the absence of copper but not in its presence. Growth on methanol in the presence of copper, however, occurred in the presence of MOPS buffer that stabilized pH. Examination of transcriptome profiles, NADH/NAD ratios, and formate production indicates that growth disruption in the presence of copper was likely caused by a confluence of (1) overgeneration of reducing power from methanol oxidation; (2) formate accumulation leading to acidification of the growth medium and inactivation of formate dehydrogenase; and (3) lack of expression of oxidative stress defence genes. Finally, transcriptomic analysis showed that expression of two putative siderophore gene clusters (one on a plasmid and the other on the chromosome) was significantly controlled by both the availability of copper as well as growth substrate (i.e., methane vs. methanol). Specifically, the presence of methanol and copper significantly repressed the expression of these siderophore gene clusters, suggesting that these metallophores play a key role in facilitating methanotrophic growth.

## Graphical abstract

*Methylosinus trichosporium* OB3b can grow on methanol in the absence but not presence of copper due: (1) overgeneration of reducing power from methanol oxidation; (2) formate accumulation leading to acidification of the growth medium and inactivation of formate dehydrogenase; and (3) lack of expression of potential oxidative stress defence genes.

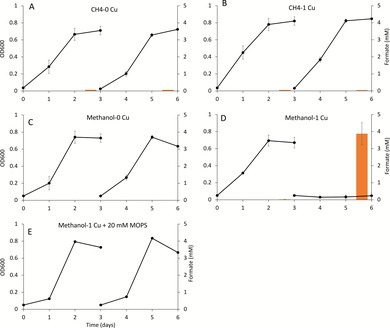

## 1 Introduction

**FIGURE 1:**
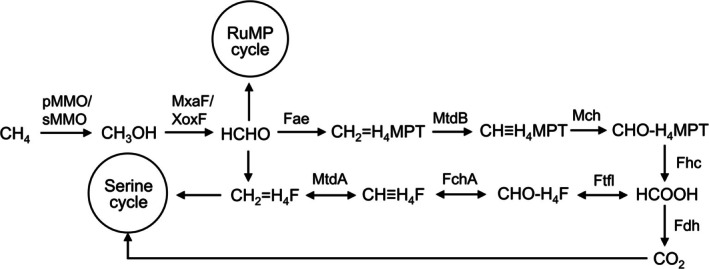
General methane metabolic pathway in aerobic methanotrophs and the enzymes involved. Fae, formaldehyde activating enzyme; FchA, methenyltetrahydrofolate cyclohydrolase; Fdh, formate dehydrogenase; Fhc, tetrahydromethanopterin formyltransferase; Ftfl, formatetetrahydrofolate ligase; Mch, methenyltetrahydromethanopterin cyclohydrolase; MtdA, NADP‐dependent methylenetetrahydrofolate dehydrogenase; MtdB, methylene tetrahydrofolate dehydrogenase; MxaF/XoxF, calcium/lanthanum‐dependent methanol dehydrogenase; pMMO/sMMO, Particulate/soluble methane monooxygenase.

Aerobic methanotrophs are a group of microbes that can use methane as their sole carbon and energy source (Semrau et al. 2010). These microbes oxidize methane to CO_2_ with methanol, formaldehyde, and formic acid as key intermediates as shown in Figure 1 (Semrau et al. 2010, 2018; Kalyuzhnaya et al. 2019). The first step of methane oxidation, the conversion of methane to methanol, is catalysed by the methane monooxygenase (MMO). Two forms of methane monooxygenase are known to exist, that is, the membrane‐bound or particulate methane monooxygenase (pMMO) and the cytoplasmic or soluble methane monooxygenase (sMMO) (Colby et al. 1977; Nguyen et al. 1994; Choi et al. 2003; Banerjee et al. 2019; Koo and Rosenzweig 2021; Tucci and Rosenzweig 2024). pMMO is used by most aerobic methanotrophs and its activity is strongly dependent on copper (Nguyen et al. 1994, 1998; Lieberman and Rosenzweig 2005; Martinho et al. 2007; Balasubramanian et al. 2010; Semrau et al. 2018; Kalyuzhnaya et al. 2019). A small number of characterized methanotrophs have both pMMO and sMMO (Banerjee et al. 2019), and in these methanotrophs, the expression and activity of the two forms of MMO are also dependent on copper. That is, sMMO expression/activity is only observed in the absence of copper, while pMMO expression/activity increases with increasing copper (Stanley et al. 1983; Burrows et al. 1984; Choi et al. 2003). The product of MMO‐catalysed methane oxidation, methanol, is further oxidized to formaldehyde by methanol dehydrogenase (MeDH) that also has two forms—the calcium‐dependent MxaF and lanthanum/cerium‐dependent XoxF. Formaldehyde can be used as the initial substrate for carbon assimilation via the ribulose monophosphate (RuMP) pathway or combined with tetrahydrofolate (H_4_F) to enter the serine cycle. Formaldehyde can also be oxidized to formate via the tetrahydromethanopterin (MPT) pathway. Formate is then oxidized to CO_2_ to generate reducing equivalents or through a multi‐step process can be converted to methylene tetrahydrofolate, eventually entering the serine cycle for carbon assimilation (Semrau et al. 2010) (Figure 1).

As methanol is the immediate product of methane oxidation, it would seem trivial for methanotrophs to utilize methanol as their sole carbon and energy source. Indeed, one could speculate that using methanol as a carbon source as opposed to methane would be more advantageous as methanol would liberate more electrons that could be used to generate a proton motive force for ATP production. That is, four net electrons are released from the oxidation of methane to carbon dioxide, while six net electrons are released from the oxidation of methanol to carbon dioxide. More specifically, oxidation of methane to methanol with concomitant reduction of dioxygen requires four electrons, two that come from carbon in methane and two from an exogenous reductant. It is well known that the exogenous reductant used by sMMO is NADH, while for pMMO there are currently three competing models where electrons are transferred from the methanol dehydrogenase in some fashion, that is, either directly (direct coupling), via a series of cytochromes (redox‐arm), or via ubiquinone (uphill electron transfer) with varying amounts of evidence supporting each pathway (Choi et al. 2003; Banerjee et al. 2019; Tucci and Rosenzweig 2024).

Many described methanotrophs have been reported to be able to use methanol as a sole carbon and energy source (Table S1). However, the physiology and metabolism of methanol‐grown methanotrophs can be very different than when grown on methane. For example, when *Methylotuvimicrobium alcaliphilum* 20Z was grown on methanol, significant formate accumulation (34 mM, ~25% of the methanol consumed) was observed, but such formate accumulation was not detected when this strain was grown on methane (Nguyen et al. 2020). In addition, the C_1_‐carbon assimilation pathway in *Mtv. alcaliphilum* 20Z is different when grown on methane versus methanol. That is, methane‐grown cells were found to use the RuMP cycle along with Embden‐Meyerhof‐Parnas (EMP) and Entner–Doudoroff (ED) pathways for carbon assimilation, while methanol‐grown cells used the RuMP cycle along with the EMP pathway and serine cycle for carbon assimilation (Nguyen et al. 2020). Such metabolic shift leads to more electron consumption as the serine cycle consumes more reducing equivalents than the RuMP pathway (Anthony 1982). A similar physiological change (i.e., formate accumulation) and metabolic shift (i.e., increased carbon flux through the serine cycle) were also observed in *Methylomicrobium buryatense* 5GB1 when this methanotroph was grown on methanol versus methane (Fu et al. 2019).

On the other hand, some methanotrophs exhibit poor growth on methanol for example, *Methylocystis* sp. Rockwell, *Methylobacter tundripaludum* SV96, *Methylosinus trichosporium* OB3b (Wartiainen et al. 2006; Tays et al. 2018) (Table S1). Moreover, some methanotrophs, that is, of the *Methylocystis*, *Methylomonas*, and *Methylocaldum* genera (Table S1), cannot grow on methanol. The reason for the inhibition/lack of growth with methanol in these methanotrophs is as yet unclear but it should be noted that these strains span a broad range of phylogenetic and functional diversity (e.g., representation of both Alpha‐ or Gamma‐proteobacteria, ability to express the RuMP and/or serine cycle for carbon assimilation, as well as variable ability to express sMMO).

Although copper is well‐known to affect the physiology of aerobic methanotrophs, the combined impact of copper and growth substrate (i.e., methane vs. methanol), to the best of our knowledge, has yet to be examined. To consider the possibility of compounded effects of copper and growth substrate on methanotrophic physiology, we chose to examine *Msn. trichosporium* OB3b for several reasons: (1) *Msn. trichosporium* OB3b is perhaps the best‐characterized methanotroph, and findings here can be compared to a wealth of existing data, (2) *Msn. trichosporium* OB3b has been reported to use methanol as well as methane as a growth substrate (Farhan Ul Haque et al. 2017; Tays et al. 2018), (3) unlike *Mtv. alcaliphilum* 20Z and *Mmc. buryatense* 5GB1 that are type I methanotrophs belonging to Gamma‐proteobacteria, *Msn. trichosporium* OB3b is a type II methanotroph of the Alpha‐proteobacteria that has only the serine cycle (Matsen et al. 2013; Yang et al. 2013), simplifying examination of how carbon source affects carbon assimilation, (4) copper has a significant impact on the physiology and methane metabolism in *Msn. trichosporium* OB3b as it can express both pMMO and sMMO (Gu and Semrau 2017; Peng et al. 2023), and (5) transcriptional analyses show expression of genes involved in methanol oxidation and carbon assimilation decrease when *Msn. trichosporium* OB3b is grown on methanol versus methane (Farhan Ul Haque et al. 2017).

There is no information, however, about the impact of growth substrate (methane vs. methanol) in the presence or absence of copper on the carbon flux, metabolism, and general transcriptome of *Msn. trichosporium* OB3b. Knowledge of these aspects is of significant value as *Msn. trichosporium* OB3b is a promising platform for biosynthesis of many valuable chemicals. For example, as *Msn. trichosporium* OB3b can grow on either methanol or methane, it is an attractive strategy for methanol‐based biosynthesis of valuable compounds such as methanobactin that has significant potential as a therapeutic agent to treat Wilson Disease (Müller et al. 2018; Einer et al. 2023; Peng et al. 2024). Indeed, the addition of methanol is easier, safer, and often more cost‐efficient than using methane. For instance, methanol can be more easily added as a liquid versus methane, which must be added as a gas; methane is explosive at atmospheric concentrations between 5% and 15% (v/v), while methanol concentrations in water must be greater than 20% v/v to be flammable (typical methanol concentrations used for methanotrophic growth are on the order of 5% v/v or less (Gu and Semrau 2017)), and the cost of HPLC grade methanol (purity > 99%) is about 50% of the cost of high purity (99.99%) methane (in equimolar amounts).

Further, despite some insights provided with the examination of *Mtv. alcaliphilum* 20Z and *Mmc. buryatense* 5GB1, there is still a general knowledge gap regarding methanol metabolism in methanotrophs. For example, why are some methanotrophs unable to grow on methanol (Table S1)? For methanotrophs that have been shown to grow on methanol, are there any cumulative toxic effects from doing so (e.g., buildup of formate) that ultimately inhibit growth? Does copper have an impact on methanol metabolism in methanotrophs? Here, we address these questions by investigating the impact of copper on the physiology and transcriptome of *Msn. trichosporium* OB3b grown with methanol versus methane.

## 2 Materials and Methods

### 2.1 Growth Conditions

*Msn. trichosporium* OB3b was grown in nitrate mineral salt (NMS) medium (Whittenbury et al. 1970) or NMS medium without Fe‐EDTA (iron‐limited NMS medium) with or without copper (1 μM as CuCl_2_) and/or a buffer to control pH that does not change copper speciation (20 mM 3‐(N‐morpholino)propanesulfonic acid (MOPS)) (Mash et al. 2003). Methane and air were added at a methane‐to‐air ratio of 1:2. Cultures were incubated in the dark at 30°C. When methanol was used as the carbon source, HPLC grade methanol (> 99%) was added to NMS medium at a concentration of 0.25% (v/v). Liquid cultures were grown in 250‐ml sidearm Erlenmeyer flasks with 20 mL NMS medium with shaking at 200 rpm. Growth was monitored non‐invasively by using the side arm to measure the optical density at 600 nm (OD_600_) with a Genesys 20 visible spectrophotometer (Spectronic Unicam, Waltham, MA). Triplicate biological cultures were prepared for all experimental conditions.

### 2.2 RNA Extraction, Sequencing, and Data Analysis

RNA was extracted from active growing cultures at middle to late exponential phase. RNA extraction was performed with a bead‐beating procedure followed by column purification using an RNeasy Mini Kit (Qiagen, Hilden, Germany) as described before (Peng et al. 2017). Genomic DNA was removed from the column with RNase‐free DNase (Qiagen, Hilden, Germany) treatment. Purified RNA was quantified using a NanoDrop 1000 Spectrophotometer (Thermo Scientific, Wilmington, DE).

RNA libraries were prepared and paired‐end sequenced on the NovaSeq system at the Advanced Genomics Core at the University of Michigan. Read quality was assessed with FastQC and the adapter sequence was removed using Cutadapt (Martin 2011). Total reads of the collected cDNA ranged from 6.8 × 10^7^ to 1.1 × 10^8^ per sample, with 66%–92% of reads assigned to protein encoding regions of the genome of *Msn. trichosporium* OB3b (Table S2). Reads were mapped against the reference genome of *Msn. trichosporium* OB3b (GenBank accession number: GCA_002752655.1) using STAR (Dobin et al. 2013). Counting of the reads was performed using featureCounts (Liao et al. 2014). Analysis of the differential gene expression based on the reads counting was performed with DESeq2 (Love et al. 2014). antiSMASH was used to identify if genes involved in secondary metabolites biosynthesis (e.g., methanobactin and potential siderophores) were differentially expressed in *Msn. trichosporium* OB3b (Blin et al. 2025) when grown on methane versus methanol.

### 2.3 cDNA Synthesis and Reverse Transcription PCR (RT‐PCR)

cDNA was synthesized from 200 ng total RNA using SuperScript III Reverse Transcriptase (Invitrogen, Carlsbad, CA) following manufacturer's instructions. RT‐qPCR was performed with 1 μL cDNA to determine the relative expression of genes involved in methane oxidation (*pmoA*, *mmoX*) and copper uptake (*mbnA*) (Table S3) in *Msn. trichosporium* OB3b under different growth conditions. The RT‐qPCR was performed using the iTaq Universal SYBR Green Supermix (Bio‐Rad, Hercules, CA) with 96‐well PCR plates on a CFX Connect real‐time PCR detection system (Bio‐Rad, Hercules, CA) with the following program: 95°C for 5 min, followed by 30 cycles of 95°C for 30 s, 56°C for 30 s and 72°C for 30 s, and final extension at 72°C for 5 min.

### 2.4 Formate Analysis

The supernatant of *Msn. trichosporium* OB3b cultures grown under different conditions was collected at the late exponential phase. The supernatant was collected from 1 mL culture and centrifuged at 13,000 rpm for 5 min. Formate in the supernatant was analysed using a Dionex Integrion HPIC System (ThermoFisher) equipped with a Dionex IonPac analytical column (AS‐HC, 2 × 250 mm). A three‐step gradient profile was used, consisting of 10 mM KOH for 4 min, 10–40 mM KOH for 16 min, and then 40–10 mM KOH for 1.5 min.

### 2.5 NADH and NAD Analyses

NADH and NAD levels were measured in *Msn. trichosporium* OB3b at the mid‐exponential growth phase (OD_600_ ~ 0.25). NADH and NAD analysis were performed using NAD/NADH Assay Kit (Sigma‐Aldrich) following the manufacturer's protocol.

### 2.6 Transmission Electron Microscopy (TEM) Imaging

*Msn. trichosporium* OB3b cells grown under different conditions were fixed in 2.5% glutaraldehyde in 0.1 M Sorensen's buffer. The fixed samples were then stained with uranyl acetate and lead citrate. The sections were examined using a JEOL JEM 1400 Plus TEM microscope equipped with a 2 k CMOS camera and a quick release room temperature retainer EM‐11610 QR1 of ±20° tilt. The size of cells and distance between intracytoplasmic membranes (ICMs) were determined using ImageJ. For each growth condition, 20 cells were randomly selected to determine the average cell size and ICM distance.

### 2.7 Biomass‐Associated Copper and Iron Analyses

Biomass of *Msn. trichosporium* OB3b grown under different copper concentrations was collected at the late exponential phase. Biomass was collected from 10 mL culture by centrifuge at 5000 rpm for 5 min. Copper and iron associated with the biomass were measured using an inductively coupled plasma mass spectrometer (ICP‐MS, Agilent Technologies, Santa Clara, CA) as described earlier (Kalidass et al. 2015). Briefly, the collected cells were washed with PBS buffer (without copper or iron) and then digested in 35% nitric acid at 95°C for 2 h. The suspensions were mixed every 30 min by inverting the tubes 5 times. The digested cell suspensions were diluted with PBS buffer to achieve a concentration of 2% nitric acid. The determined metal content was normalized by protein concentration, which was converted from cell density as OD_600_ (i.e., 1 OD_600_ ≈ 10^8^ cells/ml ≈ 0.02 mg total protein/ml).

### 2.8 Siderophore Analysis

The presence of putative siderophores in the spent medium of *Msn. trichosporium* OB3b grown under different conditions was analysed using the chrome azurol S (CAS) assay method as (Schwyn and Neilands 1987). Briefly, *Msn. trichosporium* OB3b was grown on methane or methanol in iron‐limited NMS medium with or without copper and MOPS. The supernatant was collected at the stationary growth phase. 150 μL supernatant was mixed with 50 μL CAS shuttle solution and incubated at room temperature for 15 min. Positive and negative control reactions were prepared by mixing 150 μL iron‐limited NMS medium and 50 μL CAS shuttle solution with 10 μM desferrioxamine b (final concentration in the reaction) and NMS medium, respectively.

## 3 Results

### 3.1 Growth of *Msn. Trichosporium* OB3b With Methanol

**FIGURE 2:**
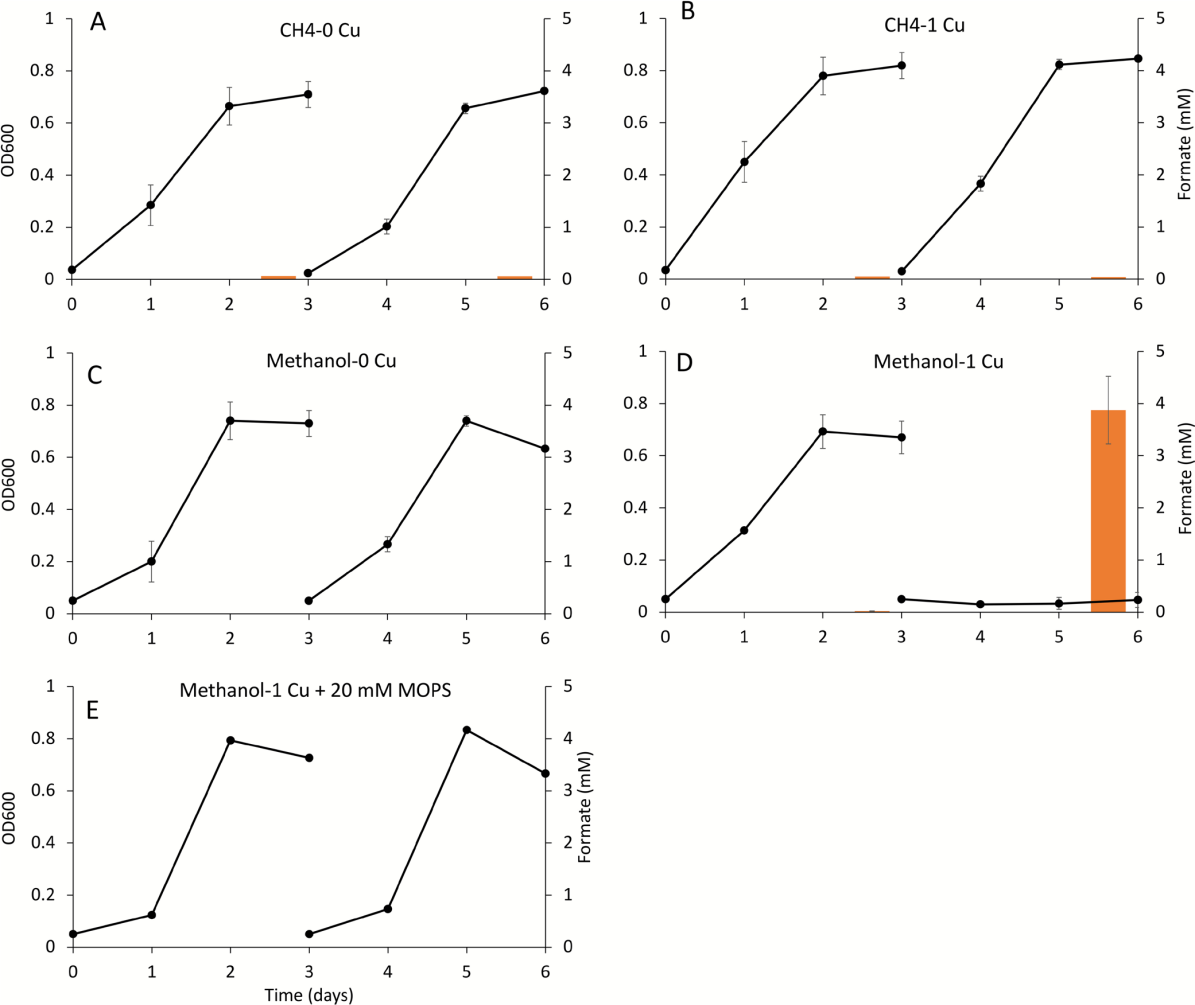
Growth and consecutive transfer of *Msn. trichosporium* OB3b grown with methanol (with and without 20 mM MOPS) in the absence of copper (0 Cu), in the presence of 1 μM copper (1 Cu), and formate accumulation (orange bar chart, measured at the last day of each growth cycle) under these growth conditions. Error bars indicate standard deviations from triplicate biological replicates.

As expected, *Msn. trichosporium* OB3b was able to grow on methane when repeatedly cultured in either the presence or absence of copper (final OD_600_ 0.6–0.8 after 3 days incubation; Figure 2). No significant formate accumulation (< 0.06 mM) was detected under these growth conditions (Figure 2), and the pH of the growth medium was 6.8 at both the beginning and the end of incubation. A similar growth pattern was observed when *Msn. trichosporium* OB3b was incubated with methanol in the absence of copper (Figure 2). Conversely, *Msn. trichosporium* OB3b was able to initially grow on methanol in the presence of 1 μM copper, but no growth was observed when *Msn. trichosporium* OB3b was transferred a second time to fresh NMS medium with methanol +1 μM copper. Interestingly, no formate accumulation or pH shift was observed in the first growth cycle, but significant formate accumulation (~3.8 mM) and a pH shift from 6.8 to 5.5 was observed in the second growth cycle. When extra buffer (20 mM MOPS) was added, *Msn. trichosporium* OB3b was able to repeatedly grow with methanol +1 μM copper and no formate accumulation or pH shift was observed (Figure 2).

Further analyses found that the growth rate of *Msn. trichosporium* OB3b on methane without copper was 2.04–2.13 d^−1^, significantly lower than growth on methane plus copper (2.51–2.56 d^−1^) (*p* ≤ 10^−4^). The growth rate of *Msn. trichosporium* OB3b on methanol in the absence of copper (1.33–1.67 d^−1^) as well as on methanol plus copper for the first cycle only (1.84 d^−1^) was significantly less than corresponding growth on methane (*p* ≤ 10^−3^ for growth on methane versus methanol in the absence of copper, *p* < 10^−4^ for growth on methane versus methanol in the presence of copper). Adding MOPS to *Msn. trichosporium* OB3b grown on methanol and in the presence of copper resulted in growth similar to that observed for *Msn. trichosporium* OB3b grown on methanol in the absence of copper (growth rate = 1.34, *p* > 0.2) (Table S4).

### 3.2 Copper and Iron Uptake of *Msn. Trichosporium* OB3b Grown With Methanol

**FIGURE 3:**
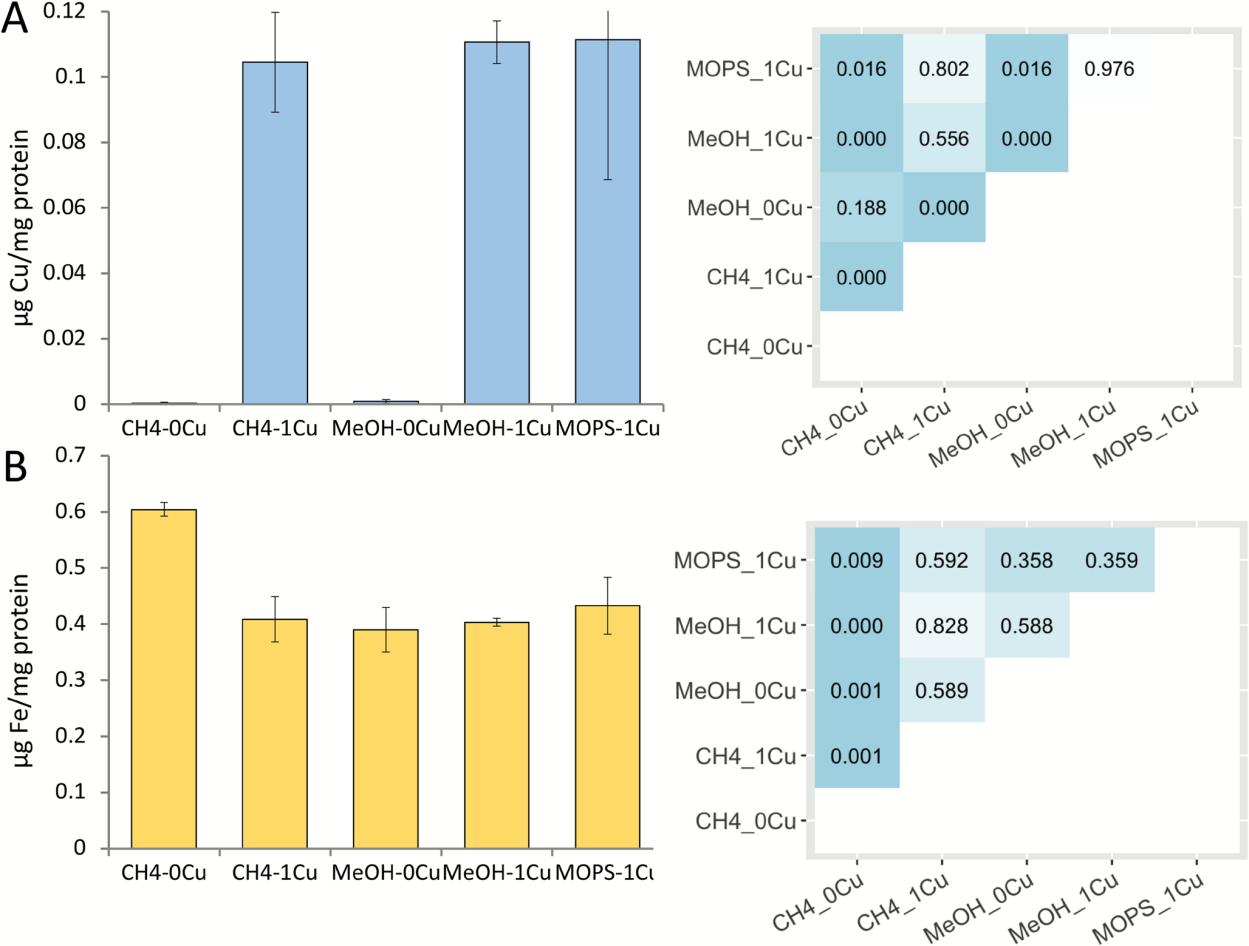
Biomass associated copper (A) and iron (B) analysis in *Msn. trichosporium* OB3b cells either grown with methane or methanol (with and without 20 mM MOPS), in the presence/absence of copper (left panel). Error bars indicate standard deviations from triplicate biological replicates. Heatmap showing *p* values based on *t*‐test from pairwise comparisons. The *p* values less than 0.05 are highlighted in blue (right panel).

The amount of copper associated with biomass was significantly higher when *Msn. trichosporium* OB3b was grown in the presence of copper with either methane or methanol—with and without MOPS—as compared to when no copper was added (i.e., ~0.11 μg Cu/mg protein vs. < 0.001 μg Cu/mg protein, respectively) (*p* < 10^−4^) (Figure 3A). No significant differences in biomass‐associated copper were found when growth substrate (methane vs. methanol) was varied either in the absence or presence of copper, nor did the presence of MOPS have any effect.

The amount of iron associated with biomass was significantly higher in *Msn. trichosporium* OB3b grown with methane without copper as compared to all other tested conditions (i.e., ~0.6 μg Fe/mg protein vs. ~0.4 μg Fe/mg protein, *p* < 0.01) (Figure 3B). No significant difference in iron associated with biomass was observed in *Msn. trichosporium* OB3b grown on methane plus copper as compared to growth on methanol with or without copper or in the presence of MOPS (Figure 3B).

### 3.3 NADH/NAD Analysis of *Msn. Trichosporium* OB3b With Methane or Methanol as the Sole Growth Substrate

**FIGURE 4:**
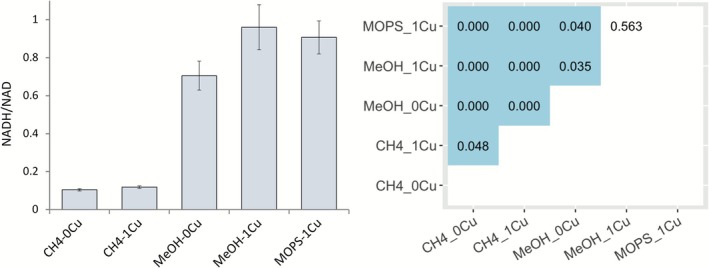
NADH/NAD analysis in cells of *Msn. trichosporium* OB3b either grown with methane or methanol (with and without 20 mM MOPS), in the presence/absence of copper. Error bars indicate standard deviations from triplicate biological replicates (left panel). Heatmap showing *p* values based on *t*‐test from pairwise comparisons. The *p* values less than 0.05 are highlighted in blue (right panel).

The NADH/NAD ratio in *Msn. trichosporium* OB3b grown on methane with and without copper was 0.1 ± 0.006 and 0.12 ± 0.006, respectively (Figure 4), and the increase, although slight, could be considered marginally significant (*p* = 0.048). For methanol‐grown cultures of *Msn. trichosporium* OB3b, the NADH/NAD ratio was ~10 times higher in the presence of copper versus methane‐grown cells plus copper (i.e., 0.96 vs. 0.12, *p* < 10^−4^). For *Msn. trichosporium* OB3b grown on methanol without copper, NADH/NAD was ~30% lower than that observed when *Msn. trichosporium* OB3b was grown on methanol plus copper (i.e., 0.70 vs. 0.96; *p* = 0.035). The NADH/NAD ratio for *Msn. trichosporium* OB3b grown with methanol plus copper with MOPS was found to be similar to that of *Msn. trichosporium* OB3b grown without MOPS (i.e., 0.90 vs. 0.96, *p* = 0.5, Figure 4).

### 3.4 TEM Images of *Msn. Trichosporium* OB3b With Methane or Methanol as the Sole Growth Substrate

*Msn. trichosporium* OB3b grown on methanol without copper had an approximately 20% smaller cross‐sectional area than *Msn. trichosporium* OB3b grown on methane or methanol plus copper, as well as on methane without copper (0.46 μm^2^ vs. 0.59–0.62 μm^2^, *p* = 0.02). Intracytoplasmic membranes (ICMs) tightly aligned parallel to the cytoplasmic membrane were observed in *Msn. trichosporium* OB3b grown with methane plus copper (Figure S1). In approximately half of the examined TEM images of *Msn. trichosporium* OB3b grown on methanol and copper (10 of 20), ICMs were also observed, but such ICMs were poorly organized and had greater spacing (i.e., 18 vs. 43 nm, *p* < 10^−6^). In the other half, no ICMs were observed, similar to that observed in TEM images of *Msn. trichosporium* OB3b grown either in the presence of methane or methanol without copper (Figure S1).

### 3.5 Transcriptional Analysis of Copper‐Regulated Genes and Genes Involved in Copper Uptake and Storage

**FIGURE 5:**
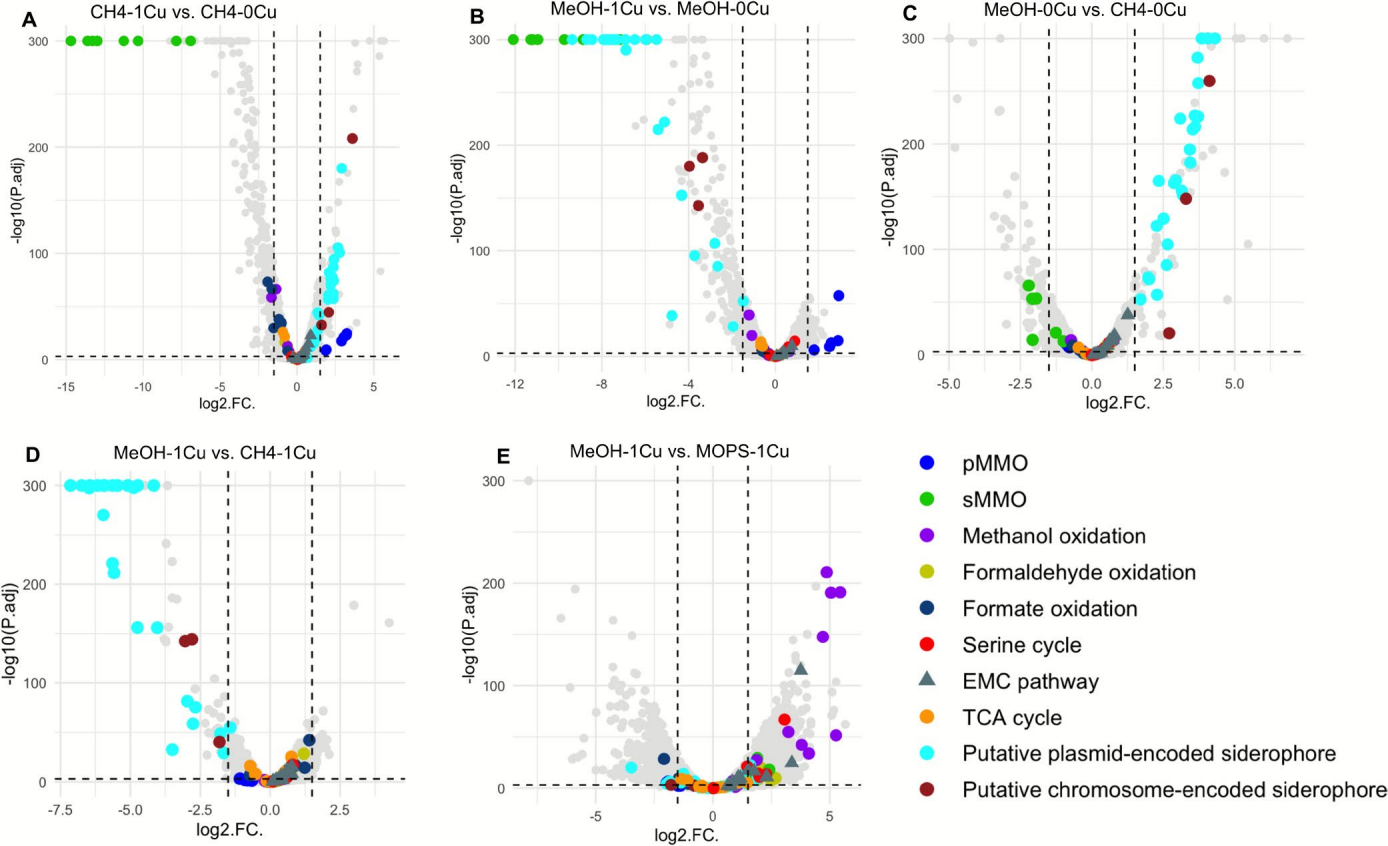
Volcano plots showing differential gene expression of *Msn. trichosporium* OB3b growing with methane or methanol (with and without 20 mM MOPS), in the presence/absence of 1 μM copper. The y‐axis represents the negative log10 of the adjusted *p* value (−log10(P.adj)). Significance thresholds, indicated by grey dashed lines, are set at an absolute log2‐fold change value (log2.FC) ≥ 1.5 and an adjusted *p* value ≤ 0.001. The differentially expressed genes with −log10(P.adj) value < 300 were plotted using −log10(P.adj) value = 300.

The transcriptomic analysis result with differential gene expression under all tested growth conditions is shown in Sheets 1–7 (Supporting Information). The overall transcriptome profile of *Msn. trichosporium* OB3b grown under different conditions was distinct from each other (Figure S2). As expected, pMMO gene (*pmoCAB*) expression was significantly up‐regulated when *Msn. trichosporium* OB3b was grown in the presence versus absence of copper when either methane or methanol was provided as the sole growth substrate (log_2_Fold Change [log_2_FC]: 2.5 ~ 3, *p* < 0.001) (Figure 5A,B, SheetS 2–4 (Supporting Information)). Further, when *Msn. trichosporium* OB3b was grown in the presence of methanol, copper and MOPS, transcription of *pmo* genes was similar to that grown with methane or methanol in the presence of copper (log_2_FC = 0.6–1) (Figure 5E, Sheet 7 (Supporting Information)). Conversely, expression of genes encoding polypeptides of sMMO (*mmoXYZDBC*) was repressed in the presence of copper regardless if *Msn. trichosporium* OB3b was provided methane or methanol, as well as if MOPS was added in conjunction with methanol (log_2_FC value: −8 to −15, *p* < 0.001) (Figure 5A–E, Sheets 3–7 (Supporting Information)). Expression of *mbn* genes encoding for synthesis of the key copper binding compound methanobactin was, as found earlier (Gu and Semrau 2017), down‐regulated in the presence versus absence of copper if *Msn. trichosporium* OB3b was grown with methane (log_2_FC value: −2 to −5, *p* < 0.001) (Figure S3). Interestingly, here we show for the first time that expression of methanobactin synthesis genes was also downregulated in the presence versus absence of copper when *Msn. trichosporium* OB3b was grown with methanol as the sole growth substrate (Figure S3). Finally, when *Msn. trichosporium* OB3b was grown in the presence of copper, methanol and MOPS, the transcription of methanobactin synthesis genes was similar to cultures grown with methanol plus copper (log_2_FC = 0.2–1.3) (Figure S3). Expression of putative copper efflux genes (*cus*) as well as known copper storage proteins genes (*csp123*) was similar under all tested growth conditions (Figure S3, Sheet 1 (Supporting Information)).

### 3.6 Transcriptional Analyses of Genes Involved in Carbon Transformation

To investigate the possible mechanism(s) underlying the observed growth inhibition and formate accumulation of *Msn. trichosporium* OB3b when grown in the presence of methanol and copper, transcription of genes involved in carbon metabolism was examined in more detail. The expression level of the genes for methanol oxidation (*mxa* gene cluster and *xoxF*), formaldehyde oxidation (e.g., *fae* encoding for formaldehyde‐activating enzyme, *ftfL* encoding formate–tetrahydrofolate ligase, and *fhc* encoding formylmethanofuran dehydrogenase, Sheet 2 (Supporting Information)), EMP pathway (e.g., *phaAB* encoding for acetyl‐CoA C‐acetyltransferase and acetoacetyl‐CoA reductase), and the serine cycle (e.g., *gly* encoding for serine hydroxymethyltransferase, Supporting Information) did not significantly change when methane or methanol was provided in the presence or absence of copper. However, when *Msn. trichosporium* OB3b was grown in the presence of methanol and copper, adding MOPS significantly decreased the expression of these genes (Figure 5E, Sheet 7 (Supporting Information)). Transcription of genes for the tricarboxylic acid (TCA) cycle (e.g., *sdh* encoding for succinate dehydrogenase, Supporting Information) and formate oxidation (*fdh* encoding formate dehydrogenase) was similar under all tested conditions (Figure 5A–E, Sheets 3–7 (Supporting Information)).

### 3.7 Transcriptional Analysis of Two Potential Siderophore Biosynthesis Gene Cluster Encoded by *Msn. Trichosporium* OB3b

The genome of *Msn. trichosporium* OB3b contains three plasmids, pOB3b1, pOB3b2, and pOB3b3 (Heil et al. 2017). One of the plasmids—pOB3b3—carries a gene cluster that was identified by antiSMASH as being putatively involved in siderophore biosynthesis, transport, and regulation. That is, this gene cluster contains nonribosomal peptide synthetase (NRPS) and polyketide synthase (PKS) encoding genes for assembly, (anti)‐sigma and TonB‐dependent receptor genes for uptake/regulation (*fpv* genes), efflux transporter genes (e.g., efflux RND transporter genes), and modification genes (e.g., aminotransferase, oxygenase genes) for a putative siderophore (Figure S4). This gene cluster was found to be most similar to the crochelin biosynthesis gene cluster found in *Azotobacter chroococcum* via ANTI‐SMASH analyses (Figure S4) (Baars et al. 2018). Transcriptomic data showed this siderophore gene cluster was up‐regulated when *Msn. trichosporium* OB3b was grown in the presence versus absence of copper with methane as the sole growth substrate (Figure 5A, Sheets 2, 3 (Supporting Information); log_2_FC value: 1.5 ~ 3, *p* < 0.001). However, expression of these genes was significantly repressed in the presence versus absence of copper when methanol was provided as the growth substrate (log_2_FC value: −10 to −1.5, *p* < 0.001) (Figure 5B, Sheet 5 (Supporting Information)). Further, these genes were repressed when *Msn. trichosporium* OB3b was grown in the presence of methanol and copper as compared to growth in the presence of methane plus copper (Figure 5D, Sheet 6 (Supporting Information)). When *Msn. trichosporium* OB3b was grown in the presence of methanol, copper and MOPS, the transcription of this putative siderophore gene cluster was similar to that grown with methanol plus copper (Figure 5E, Sheet 7 (Supporting Information)). Another putative siderophore gene cluster was found via antiSMASH in the chromosome of *Msn. trichosporium* OB3b (Figure S5), and also responded similarly to copper and carbon source as the plasmid‐encoded metallophore (Figure 5, Sheets 2–7 (Supporting Information)). To independently verify differences found via whole‐cell transcriptomics, RT‐qPCR was also performed for a handful of genes involved in methane oxidation and copper uptake, that is, *pmoA*, *mmoX*, and *mbnA*. Expression of these genes using RT‐qPCR was found to be in agreement with that for more broad transcriptomic data (Figure S6). In line with the transcriptomic analysis of siderophore gene expression (Supporting Information—Set 2), the CAS assay showed siderophore production in *Msn. trichosporium* OB3b grown in iron‐limited NMS medium with methane (with and without copper) or methanol (without copper), but not in cultures of *Msn. trichosporium* OB3b grown on methanol with copper (Figure S7).

## 4 Discussion

Methanotrophic growth on methanol ostensibly generates 50% more reducing equivalents as compared to growth on methane. Therefore, when changing the carbon source from methane to methanol, methanotrophs likely must regulate their metabolism to accommodate for the increased amount and rate of production of these reducing equivalents. Failure in doing so can cause redox imbalance, leading to cellular damage and/or mortality. For example, redox imbalance caused by overgeneration of NADH is harmful as NADH competes with NAD for binding to many enzymes carrying out oxidation, thereby inhibiting their activity (Houtkooper et al. 2010; Szenk et al. 2017). Moreover, release of excessive electrons (or increased reducing equivalent generation) is also tightly linked to oxidative stress (e.g., reactive oxygen species (ROS) generation) that can dramatically increase cell mortality (Shee et al. 2022; Yang et al. 2025).

As expected, when switching the carbon source from methane to methanol, the generation of reducing equivalents significantly increased as indicated by the increase of the NADH/NAD ratio (Figure 4). However, transcriptomic analyses show that genes involved in carbon metabolism (i.e., methanol oxidation, formaldehyde oxidation, serine cycle, and the TCA cycle) were not differentially expressed when *Msn. trichosporium* OB3b was grown with methane versus methanol in the presence or absence of copper (Figure 5D). Due to the higher net amount of electron release from methanol versus methane oxidation, methanotrophs must enhance strategies to address electron overflow under methanol‐supported growth. In the absence of copper, it is believed that the activity of electron transport proteins is proportional to the cell membrane surface area for those proteins, and ICM structure provides additional inner‐membrane surface area to increase the abundance of these proteins (Howley et al. 2023). Therefore, the formation of ICMs may play an important role in controlling electron overflow. However, TEM images show that cells grown with methanol and copper lack well‐structured ICMs as observed in the methane‐grown cells (Figure S3). The reduced amount and loose structure of ICMs of *Msn. trichosporium* OB3b grown on methanol and copper likely caused inefficient/inhibited electron transport (Szenk et al. 2017), leading to physiological stress that contributed to the lack of growth in the following transfer cultures (Figure 2D).

Unlike *Msn. trichosporium* OB3b, previous studies of methanol metabolism in *Mmc. buryatense* 5GB1 and *Mtv. alcaliphilum* 20Z indicate a shift in core metabolism. That is, in these methanotrophs, expression and utilization of a secondary carbon assimilation pathway (the serine cycle in place of the RuMP pathway) was upregulated when the carbon source was changed from methane to methanol (Fu et al. 2019; Nguyen et al. 2020). This strategy is likely in part to handle electron overflow as the serine cycle is less efficient than the RuMP pathway, that is, the serine cycle consumes rather than produces NAD(P)H (Anthony 1982). Moreover, formate accumulation was observed for both strains when growing on methanol. It would be expected that *Msn. trichosporium* OB3b accumulates formate as a regulatory strategy to reduce NAD(P)H generation when grown on methanol. However, formate accumulation was not observed in *Msn. trichosporium* OB3b grown on methanol and copper in the first growth cycle (Figure 2D). Rather, formate accumulation was observed in the following transfer cultures although there was no growth (Figure 2D). As genes for the formate dehydrogenase (Fdh) were not differentially expressed under all the tested conditions (Figure 5), our results suggest that in the second culture transfer Fdh enzymatic activity was inhibited to prevent additional reducing equivalent production from the conversion of formate to carbon dioxide, possibly by the pH shift of the cultures and/or copper itself as suggested earlier (Jollie and Lipscomb 1991).

On the other hand, adding MOPS enabled *Msn. trichosporium* OB3b to grow over multiple transfers in the presence of methanol and copper (Figure 2E). The cellular NADH/NAH with MOPS, however was not significantly different as compared to *Msn. trichosporium* OB3b grown on methanol and copper without MOPS (Figure 4). Such survival is not due to either: (1) reducing copper availability as MOPS has been shown not to bind copper, and thus does not change copper speciation (Mash et al. 2003) or to (2) enhanced activity of the formate dehydrogenase as expression of *fdh* genes was not appreciably different in the presence versus absence of MOPS (logFC = −1.0 to 1.3, *p* > 0.2; except for *fdhA* with logFC = −2.0 to 1.3, *p* < 10^−10^). The survival of *Msn. trichosporium* OB3b under this growth condition may be a consequence of MOPS buffering the growth medium to prevent significant pH shifts, enabling *Msn. trichosporium* OB3b to maintain metabolism. This hypothesis is supported by the finding that transcriptomic analyses showed genes involved in carbon metabolism (i.e., methanol oxidation, formaldehyde oxidation, serine cycle) were significantly down‐regulated in *Msn. trichosporium* OB3b when grown in the presence of methanol, copper, and MOPS as compared to methanol and copper alone (Figure 5E). Overall, our findings suggest that such changes could then protect *Msn. trichosporium* OB3b from potential oxidative damage. Interestingly, *Msn. trichosporium* OB3b can repeatedly grow on methanol in the absence of copper without formate accumulation (Figure 1C) but with an increased NADH/NAD ratio as compared to growth on methane in either the presence or absence of copper (Figure 2A,B). Such data support the conclusion that excess reductant generation alone is not sufficient to prevent growth, especially as transcriptomic analyses found no significant change in expression of genes for carbon metabolism for growth on methanol without copper as compared to growth on methanol with copper (Figure 5B).

Biosynthesis of siderophores may be another approach used by *Msn. trichosporium* OB3b to defend against metabolic stress from methanol oxidation. That is, besides iron uptake, siderophores can have other functions, for example, managing oxidative stress (Kramer et al. 2020). For example, deletion of enterobactin genes in *E. coli* disrupted its growth in minimal medium but growth was restored by adding enterobactin in the absence of iron as enterobactin can scavenge ROS produced via Fenton's reaction (Adler et al. 2014). Further, siderophores have been found to defend against oxidative stress in methylotrophs that grow on methanol (Juma et al. 2022). As shown in Figure 5B and Sheet 6 (Supporting Information), the expression of two siderophore gene clusters was upregulated when *Msn. trichosporium* OB3b was grown on methanol in the absence of copper, with growth over multiple transfers occurring (Figure 2C). However, growth of *Msn. trichosporium* OB3b was limited in the presence of methanol and copper, with the concomitant finding that expression of these siderophore gene clusters was significantly repressed (Figure 5B,D). The transcriptional regulation of siderophore gene expression is consistent with the CAS assay, showing (a) siderophore(s) was(were) indeed produced when *Msn. trichosporium* OB3b was grown on methanol without copper, but not when grown on methanol with copper (Figure S7). Thus, in *Msn. trichosporium* OB3b, the differential regulation of expression and biosynthesis of these siderophores may serve as oxidative stress defence as iron associated with biomass remained constant under all methanol‐growth conditions (Figure 3).

Interestingly, the two putative siderophore gene clusters were inversely regulated under methane‐growth conditions versus methanol‐growth conditions. That is, both siderophore gene clusters were up‐regulated in *Msn. trichosporium* OB3b grown on methane in the presence versus absence of copper w (Figure 5A,B). The reason for such differential regulation of siderophores biosynthesis under methane growth conditions is not clear, but suggests that as biomass‐associated iron was highest for *Msn. trichosporium* OB3b when grown on methane in the absence of copper (Figure 3B)—when expression of these siderophores was repressed—that this methanotroph uses an alternative, as yet uncharacterized means to collect iron when grown on methane in the absence of copper. Indeed, transcriptional analysis shows the expression of genes encoding several FecR family proteins (the regulatory protein for ferric citrate transport system) was significantly up‐regulated in methane‐grown cells in the absence of copper that may be responsible for the observed iron uptake (e.g., gene locus tag CQW49_RS21790, CQW49_RS03690, CQW49_RS13700, CQW49_RS02130, CQW49_RS18230. Sheet 3 (Supporting Information)).

Regarding the role(s) of these putative siderophores for growth in the presence of methane and copper, several possibilities exist. First, they may enhance the activity of various proteins involved in energy metabolism/electron transport, for example, various cytochromes embedded in the ICMs, and/or pMMO activity. That is, although copper is well known to affect pMMO activity, it has been reported that iron stimulates pMMO activity as well (Martinho et al. 2007) and these siderophore(s) may preferentially deliver iron to the ICM/periplasm as reported for pyoverdine (Bonneau et al. 2020). Second, it is possible that the primary function of these metallophores is not to bind iron, but some other trace metal, for example, it has been recently shown that methylolanthanin, a modified form of the siderophore rhodopetrobactin, will bind lanthanum (Juma et al. 2022). Third, these siderophores may also aid in handling redox stress under pMMO‐expressing conditions as suggested above for growth on methanol in the absence of copper.

## 5 Conclusions

In conclusion, herein we show that *Msn. trichosporium* OB3b makes significant metabolic adjustments to grow on methanol versus methane—particularly in the presence of copper—to handle oxidative stress resulting from the overflow of reducing equivalent generation. In addition, our data indicate that copper may inactivate the formate dehydrogenase, leading to a deleterious decrease in pH of the growth medium. We would like to note that systematic investigation of methanol metabolism in methanotrophs is limited, especially among Alpha‐proteobacteria methanotrophs. Although many methanotrophs have been reported able to grow on methanol, such data may be erroneous, for as we show here for *Msn. trichosporium* OB3b, growth on methanol only repeatedly occurs in the absence of copper. Indeed, we would like to note that there are contradictory statements in the literature as to whether *Msn. trichosporium* OB3b can grow on methanol, with the original characterization of this strain stating that it cannot (Whittenbury et al. 1970), while subsequent studies, including some from our own laboratory, suggest that it can (Farhan Ul Haque et al. 2017). Furthermore, it appears that the expression and biosynthesis of putative siderophores may play a critical role in enabling methanotrophic growth in the presence of copper. At this time, we do not know what metal(s) these siderophores may preferentially bind, and further research is thus needed to address this open question.

## Author Contributions

Peng Peng and Jeremy D. Semrau contributed equally to conceptualization as well as data collection, curation, analyses, and investigation. Peng Peng took the lead in drafting the initial manuscript while Jeremy D. Semrau took the lead in manuscript review and editing, as well as funding acquisition and project administration.

## Funding

This work was supported by the National Science Foundation (241864).

## Conflicts of Interest

The authors declare no conflicts of interest.

## Supporting information


**Data S1:** Supporting Information.

## Data Availability

The transcriptomic datasets generated during and/or analyzed during the current study are available at the Gene Expression Omnibus (https://www.ncbi.nlm.nih.gov/geo/) with accession number GSE310588.
