# Supplementary material for: Impact of Copper on the Physiology and Transcriptome of Methylosinus Trichosporium OB3b Grown on Either Methane or Methanol
Source: Environ Microbiol. 2026 Jan 16;28(1):e70245. doi: 10.1111/1462-2920.70245 (PMC12811070; doi:10.1111/1462-2920.70245)
Supplement: Supplementary file 1 — Data S1: Supporting Information. [file EMI-28-e70245-s001.zip › SI Submitted_Revised NO TRACK CHANGES.docx]

Table S1. Methanol growth and presence of MMO genes in methanotrophs

| **Strain** | ***pmoCAB*** | **sMMO** | **Methanol growth** | **Reference** |
| --- | --- | --- | --- | --- |
| **Gammaproteobacteria** |  |  |  |  |
| *Methylobacter tundripaludum* SV96 | Yes | No | Yes (poor to no growth) | (Wartiainen et al., 2006b) |
| *Methylocaldum szegediense* O-12 | Yes | No | No | (Eshinimaev et al., 2004) |
| *Methylocaldum marinum* S8 | Yes | Yes | Yes | (Takeuchi et al., 2014) |
| *Methylococcus capsulatus* Bath | Yes | Yes | Yes | (Prior and Dalton, 1985) |
| *Methyloparacoccus murrellii* R-49797 | Yes | No | Yes | (Hoefman et al., 2014b) |
| *Methyloglobulus morosus* KoM1 | Yes | No | Yes | (Deutzmann et al., 2014) |
| *Methylomagnum ishizawai* RS11D-Pr | Yes | Yes | Yes | (Khalifa et al., 2015) |
| *Methylomicrobium agile* A30 | Yes | No | Yes | (Whittenbury et al., 1970) |
| *Methylomicrobium album* BG8 | Yes | No | Yes | (Whittenbury et al., 1970) |
| *Methylomicrobium alcaliphilum* 20Z | Yes | No | Yes | (Nguyen et al., 2020) |
| *Methylomicrobium buryatense* 5GB1 | Yes | Yes | Yes | (Fu et al., 2019) |
| *Methylomonas albis* EbA | Yes | Yes | No | (Bussmann et al., 2021) |
| *Methylomonas lenta*  R-45377 | Yes | Yes | Yes | (Hoefman et al., 2014a) |
| *Methylomonas montana* MW1 | Yes | No | Yes | (Suleimanov et al., 2023) |
| *Methylosarcina fibrata* AML-C10 | Yes | No | Yes | (Wise et al., 2001) |
| *Methylovulum miyakonense* HT12 | Yes | Yes | Yes | (Iguchi et al., 2011) |
| *Methylohalobius crimeensis* 10Ki | Yes | No | Yes | (Heyer et al., 2005) |
| **Alphaproteobacteria** |  |  |  |  |
| *Methylocapsa acidiphila* B2 | Yes | No | Yes | (Dedysh et al., 2002) |
| *Methylocapsa aurea* KYG | Yes | No | Yes | (Dunfield et al., 2010) |
| *Methylocella silvestris* BL2 | No | Yes | Yes | (Dunfield et al., 2003) |
| *Methyloferula stellata* AR4 | No | Yes | Yes | (Vorobev et al., 2011) |
| *Methylocystis silviterrae* FT | Yes (2) | No | Yes | (Tikhonova et al., 2021) |
| *Methylocystis hirsute* CSC1 | Yes | Yes | Yes | (Lindner et al., 2007) |
| *Methylocystis parvus* OBBP | Yes (2) | No | Yes (need pre-adaption) | (Hou et al., 1979) |
| *Methylocystis* sp. strain Rockwell | Yes | No | Yes (poor growth) | (Tays et al., 2018) |
| *Methylocystis rosea* SV97 | Yes (2) | No | No | (Wartiainen et al., 2006a) |
| *Methylocystis* sp. SB2 | Yes | No | No | (Im et al., 2011) |
| *Methylocystis hydrogenophila* SC2 | Yes (2) | No | No | (Guo et al., 2025) |
| *Methylosinus sporium* NCIMB11126 | Yes | Yes | Yes | (Bowman et al., 1993) |
| *Methylosinus* sp. Ce-a6 | Yes | No | Yes | (Kato et al., 2020) |
| *Methylosinus* sp. 29/M242/M169 | Yes | Yes | Yes | (Kip et al., 2011) |

Table S2. RNA sequencing and mapping for *Msn. trichosporium* OB3b under different growth conditions

| **Condition** | **Read numbers** | **Mapped reads (%)** |
| --- | --- | --- |
| CH4-0CuA | 6.88E+07 | 91.44 |
| CH4-0CuB | 9.19E+07 | 89.34 |
| CH4-0CuC | 7.95E+07 | 87.15 |
| CH4-1CuA | 7.91E+07 | 72.67 |
| CH4-1CuB | 8.69E+07 | 74.3 |
| CH4-1CuC | 7.91E+07 | 72.67 |
| MeOH-0CuA | 1.03E+08 | 88.51 |
| MeOH-0CuB | 1.13E+08 | 88.53 |
| MeOH-1CuA | 6.87E+07 | 79.78 |
| MeOH-1CuB | 7.71E+07 | 83.61 |
| MeOH-1CuC | 8.27E+07 | 83.63 |
| MOPS-1CuA | 8.85E+07 | 65.84 |
| MOPS-1CuB | 8.46E+07 | 66.44 |

Table S3. Primers used in this study

| Targeted gene | Primer name and sequence (5’―3’)^a^ | Application | Reference |
| --- | --- | --- | --- |
| *pmoA* | pmoA_F (TTCTGGGGCTGGACCTAYTTC)  pmoA_R (CCGACAGCAGCAGGATGATG) | RT-qPCR | (Knapp et al., 2007) |
| *mmoX* | mmoX_F (TCAACACCGATCTSAACAACG)  mmoX_R (TCCAGATTCCRCCCCAATCC) | RT-qPCR | (Knapp et al., 2007) |
| *mbnA* | qmbnA_F (TGGAAACTCCCTTAGGAGGAA)  qmbnA_R (CTGCACGGATAGCACGAAC) | RT-qPCR | (Semrau et al., 2013) |
| 16S rRNA | Eub_341F (CCTACGGGAGGCAGCAG)  Eub_534R (ATTACCGCGGCTGCTGGC) | RT-qPCR | (Muyzer et al., 1993) |

^a^ Y, S, and R are the IUPAC DNA codes for the C/T, C/G, and A/G nucleobases, respectively.

Table S4. Maximum OD_600_ and growth rate of *Msn. trichosporium* OB3b under different growth conditions. NG = no growth.

| **Growth Condition** | **1^st^ cycle** | | **2^nd^ cycle** | |
| --- | --- | --- | --- | --- |
|  | **Max OD_600_** | **Growth rate** | **Max OD_600_** | **Growth rate** |
| CH4-0Cu | 0.71 ± 0.01 | 1.45 ± 0.1 | 0.72 ± 0.12 | 1.65 ± 0.1 |
| CH4-1Cu | 0.82 ± 0.01 | 1.55 ± 0.04 | 0.84 ± 0.1 | 1.65 ± 0.06 |
| Methanol-0Cu | 0.74 ± 0.07 | 1.34 ± 0.04 | 0.74 ± 0.02 | 1.34 ± 0.01 |
| Methanol-1Cu | 0.69 ± 0.06 | 1.31 ± 0.04 | NG | NG |
| MOPS-1Cu | 0.79 ± 0.01 | 1.38 ± 0.01 | 0.83 ± 0.11 | 1.4 ± 0.06 |


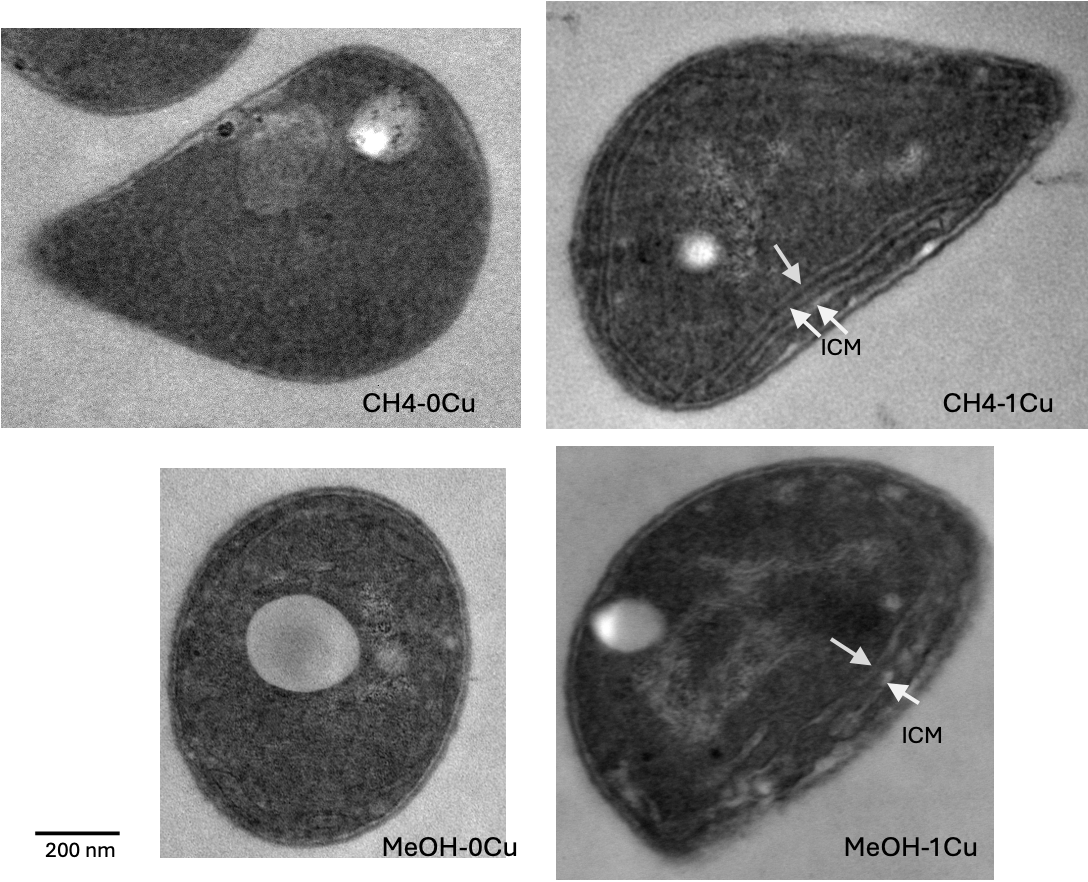


Fig S1. TEM images of *Msn. trichosporium* OB3b grown under different conditions.


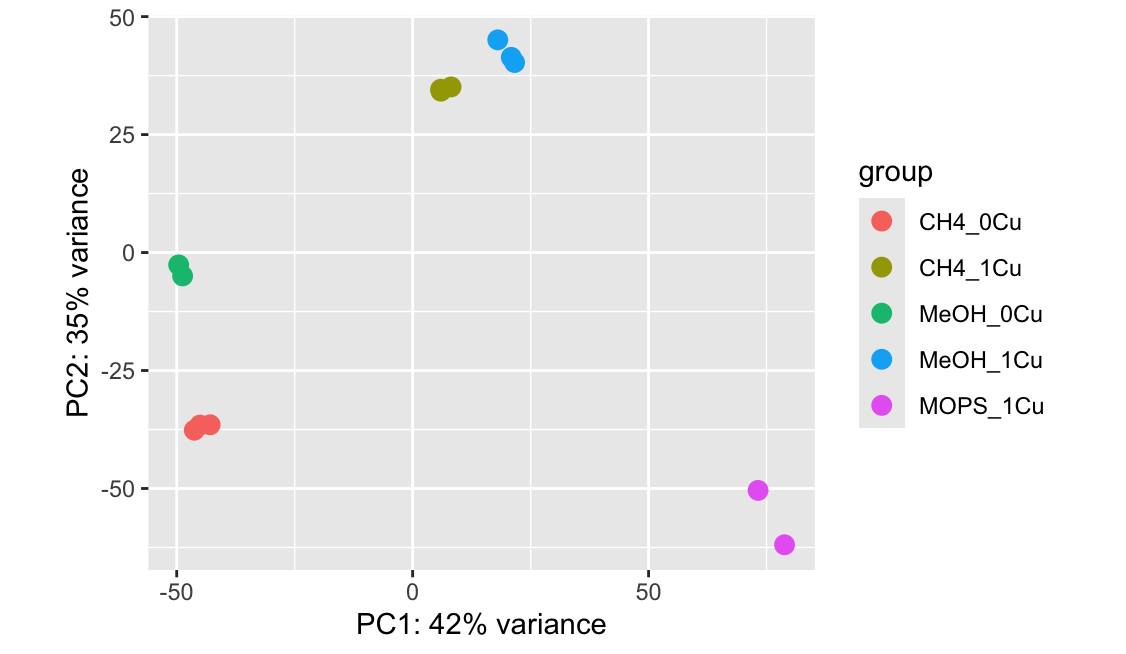


Fig S2. Principal component analysis (PCA) of the overall transcriptome of *Msn. trichosporium* OB3b grown with either methane or methanol andin the presence or absence of copper (1 μM).


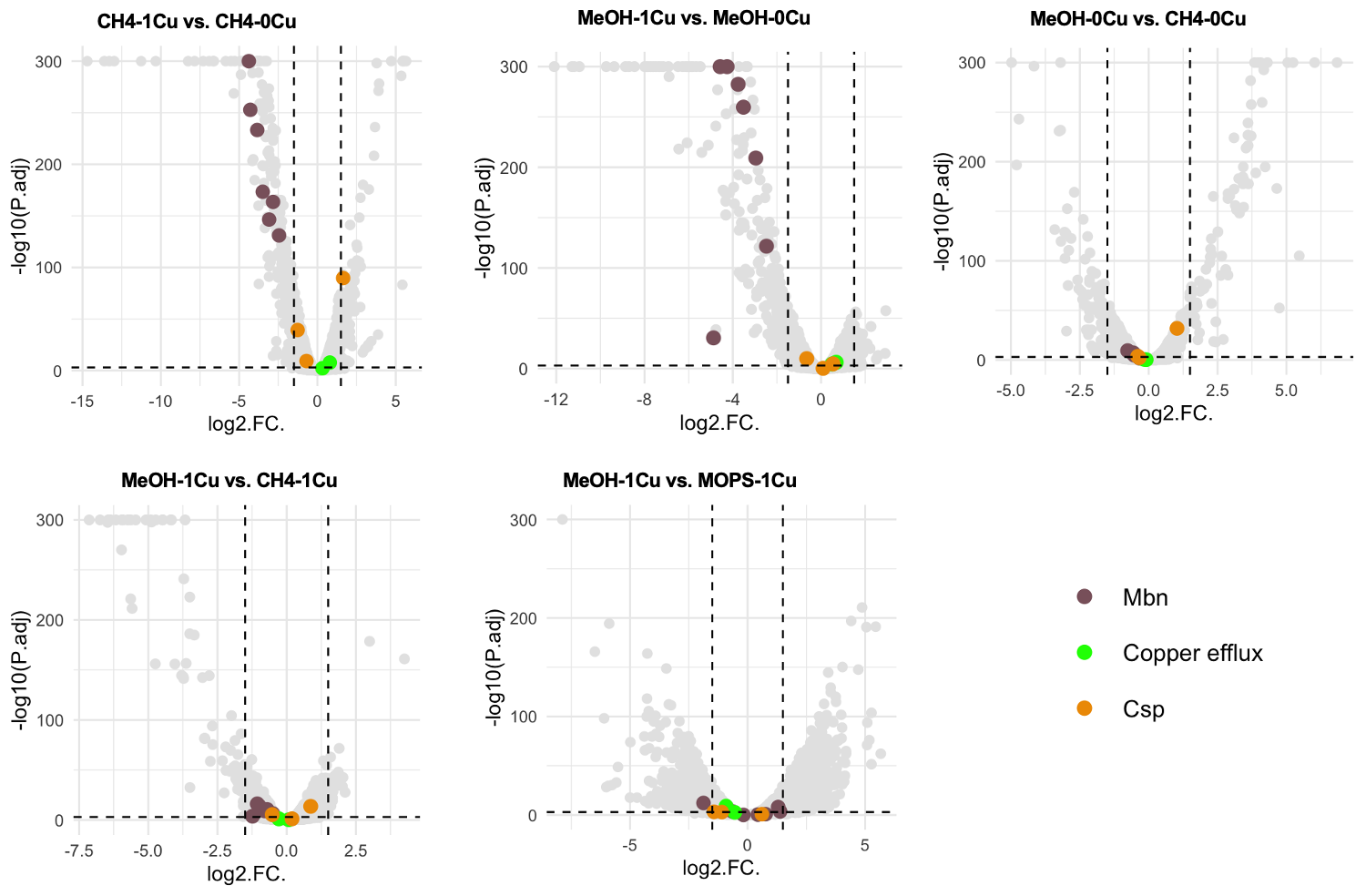


Fig S3. Volcano plots showing differential gene expression of *Msn. trichosporium* OB3b grown with either methane or methanol in the presence or absence of copper(1 μM ) and also with or without 40 mM PBS or 20 mM MOPS. The y-axis represents the negative log_10_ of the adjusted p value (−log_10_(P.adj)). Significance thresholds, indicated by grey dashed lines, are set at an absolute log_2_-fold change value (log_2_FC) ≥ 1.5 and an adjusted p value ≤ 0.001. Differentially expressed genes with −log10(P.adj) value < 300 were plotted using a −log10(P.adj) value of 300 to facilitate graphical presentation.


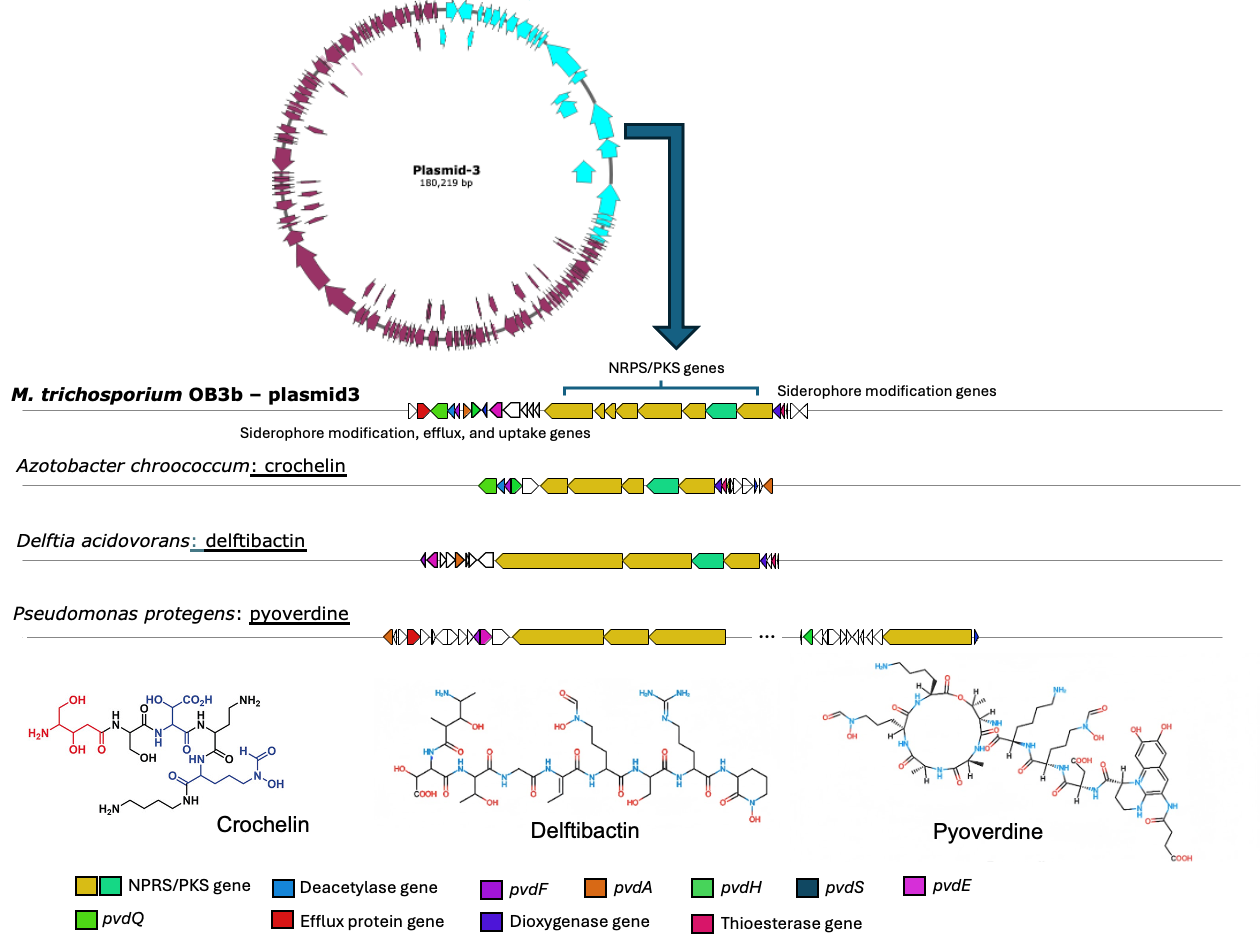


Fig S4. Comparison of the plasmid encoded putative siderophore gene clusters of *Msn. trichosporium* OB3b with other known/similar siderophore gene clusters encoded by various bacterial species. The structure of the siderophores produced by each bacterial species are shown. Genes with matching colors are interrelated with significant sequence similarity.


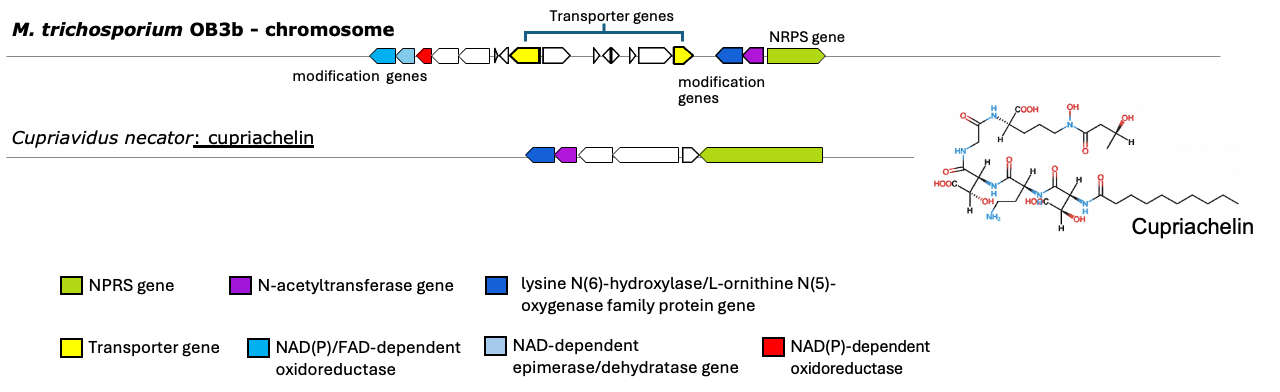


Fig S5. Comparison of the chromosome encoded putative siderophore gene clusters of *Msn. trichosporium* OB3b with the cupriachelin gene clusters encoded by *Cupriavidus necator*. The structure of the siderophores produced by each bacterial species are shown. Genes with matching colors are interrelated with significant sequence similarity.


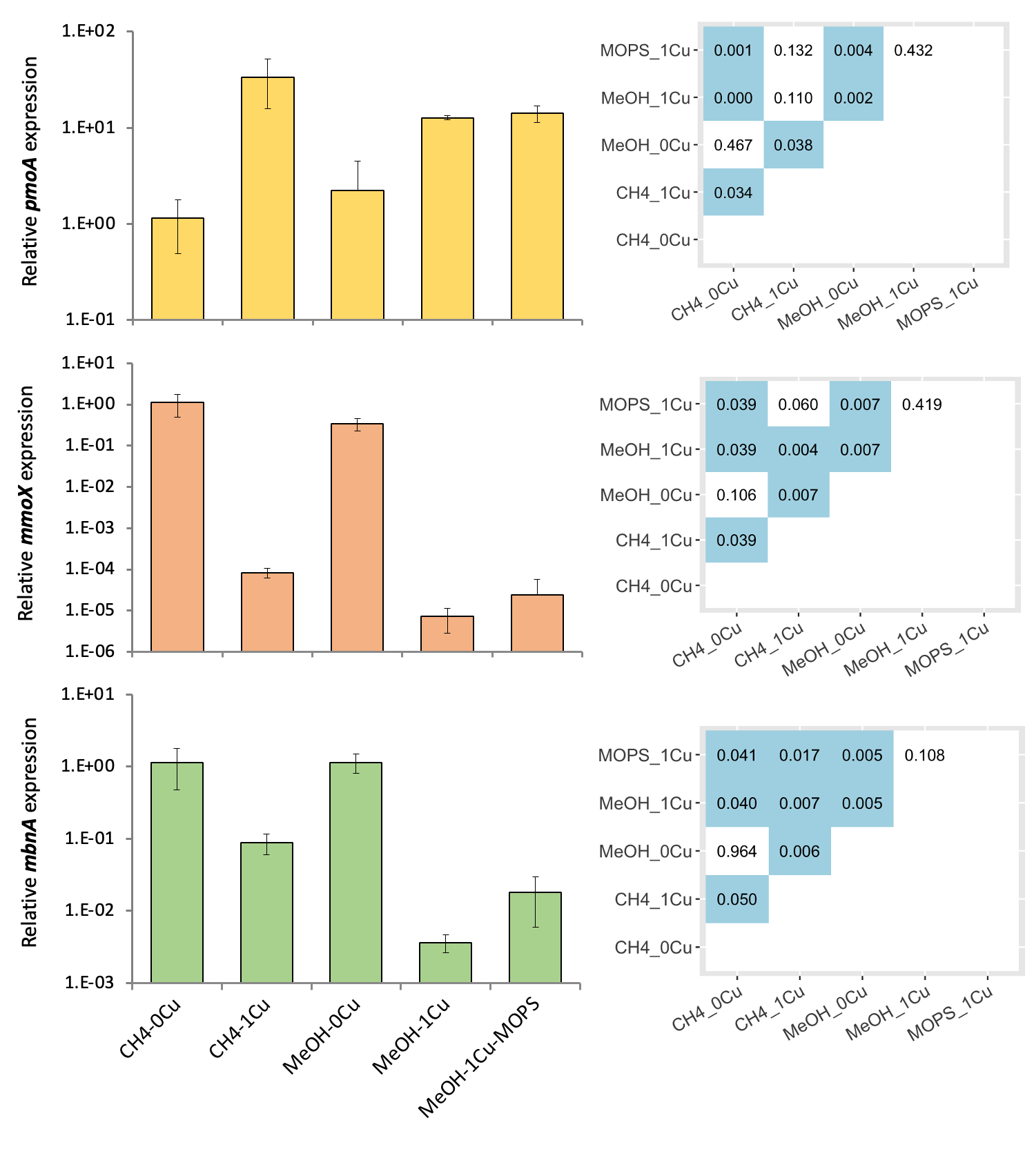


Fig S6. RT-qPCR analysis of the relative expression of *pmoA*, *mmoX*, *mbn* genes in *Msn. trichosporium* OB3b grown on methane and methanol in the presence/absence of 1 µM copper and 20 mM MOPS. Error bars indicate standard deviations from triplicate biological cultures (left panel). Heatmap showing p values based on t-test from pairwise comparisons. p values less than 0.05 are highlighted in blue.


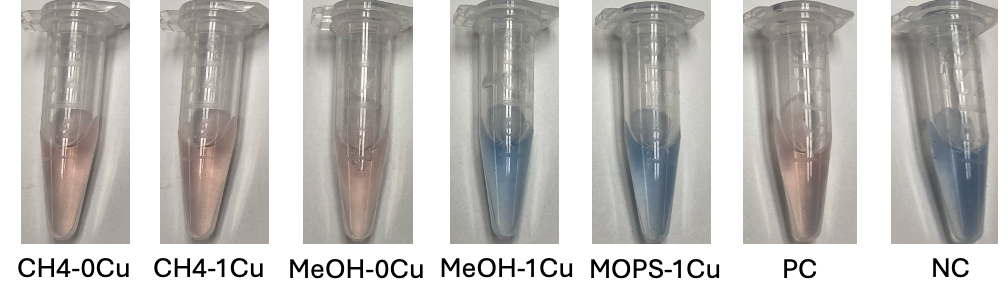


Fig S7. CAS assay of the supernatant of *Msn. trichosporium* OB3b cultures grown in iron-limited NMS medium under different conditions. The reaction of positive control (PC) contains 10 μM desferrioxamine b. Autoclaved iron-limited NMS medium was used for the negative control reaction (NC).

References

Bowman, J.P., Sly, L.I., Nichols, P.D., and Hayward, A. (1993) Revised taxonomy of the methanotrophs: description of Methylobacter gen. nov., emendation of Methylococcus, validation of Methylosinus and Methylocystis species, and a proposal that the family Methylococcaceae includes only the group I methanotrophs. *Int J Syst Evol Microbiol* **43**: 735-753.

Bussmann, I., Horn, F., Hoppert, M., Klings, K.-W., Saborowski, A., Warnstedt, J., and Liebner, S. (2021) Methylomonas albis sp. nov. and Methylomonas fluvii sp. nov.: two cold-adapted methanotrophs from the river Elbe and emended description of the species Methylovulum psychrotolerans. *Syst Appl Microbiol* **44**: 126248.

Dedysh, S.N., Khmelenina, V.N., Suzina, N.E., Trotsenko, Y.A., Semrau, J.D., Liesack, W., and Tiedje, J.M. (2002) Methylocapsa acidiphila gen. nov., sp. nov., a novel methane-oxidizing and dinitrogen-fixing acidophilic bacterium from Sphagnum bog. *Int J Syst Evol Microbiol* **52**: 251-261.

Deutzmann, J.S., Hoppert, M., and Schink, B. (2014) Characterization and phylogeny of a novel methanotroph, Methyloglobulus morosus gen. nov., spec. nov. *Syst Appl Microbiol* **37**: 165-169.

Dunfield, P.F., Khmelenina, V.N., Suzina, N.E., Trotsenko, Y.A., and Dedysh, S.N. (2003) Methylocella silvestris sp. nov., a novel methanotroph isolated from an acidic forest cambisol. *Int J Syst Evol Microbiol* **53**: 1231-1239.

Dunfield, P.F., Belova, S.E., Vorob'ev, A.V., Cornish, S.L., and Dedysh, S.N. (2010) Methylocapsa aurea sp. nov., a facultative methanotroph possessing a particulate methane monooxygenase, and emended description of the genus Methylocapsa. *Int J Syst Evol Microbiol* **60**: 2659-2664.

Eshinimaev, B.T., Medvedkova, K., Khmelenina, V., Suzina, N., Osipov, G., Lysenko, A., and Trotsenko, Y.A. (2004) New thermophilic methanotrophs of the genus Methylocaldum. *Microbiology* **73**: 448-456.

Fu, Y., He, L., Reeve, J., Beck, D.A., and Lidstrom, M.E. (2019) Core metabolism shifts during growth on methanol versus methane in the methanotroph Methylomicrobium buryatense 5GB1. *MBio* **10**: e00406-00419.

Guo, K., Heimerl, T., Hakobyan, A., Han, D., and Liesack, W. (2025) Methylocystis hydrogenophila sp. nov.—A Type IIa Methanotrophic Bacterium Able to Utilize Hydrogen as an Alternative Energy Source. *Microorganisms*.

Heyer, J., Berger, U., Hardt, M., and Dunfield, P.F. (2005) Methylohalobius crimeensis gen. nov., sp. nov., a moderately halophilic, methanotrophic bacterium isolated from hypersaline lakes of Crimea. *Int J Syst Evol Microbiol* **55**: 1817-1826.

Hoefman, S., Heylen, K., and De Vos, P. (2014a) Methylomonas lenta sp. nov., a methanotroph isolated from manure and a denitrification tank. *Int J Syst Evol Microbiol* **64**: 1210-1217.

Hoefman, S., van der Ha, D., Iguchi, H., Yurimoto, H., Sakai, Y., Boon, N. et al. (2014b) Methyloparacoccus murrellii gen. nov., sp. nov., a methanotroph isolated from pond water. *Int J Syst Evol Microbiol* **64**: 2100-2107.

Hou, C., Laskin, A., and Patel, R. (1979) Growth and polysaccharide production by Methylocystis parvus OBBP on methanol. *Appl Environ Microbiol* **37**: 800-804.

Iguchi, H., Yurimoto, H., and Sakai, Y. (2011) Methylovulum miyakonense gen. nov., sp. nov., a type I methanotroph isolated from forest soil. *Int J Syst Evol Microbiol* **61**: 810-815.

Im, J., Lee, S.W., Yoon, S., DiSpirito, A.A., and Semrau, J.D. (2011) Characterization of a novel facultative *Methylocystis* species capable of growth on methane, acetate and ethanol. *Environ Microbiol Rep* **3**: 174-181.

Kato, S., Takashino, M., Igarashi, K., and Kitagawa, W. (2020) Isolation and genomic characterization of a proteobacterial methanotroph requiring lanthanides. *Microbes Environ* **35**: ME19128.

Khalifa, A., Lee, C.G., Ogiso, T., Ueno, C., Dianou, D., Demachi, T. et al. (2015) Methylomagnum ishizawai gen. nov., sp. nov., a mesophilic type I methanotroph isolated from rice rhizosphere. *Int J Syst Evol Microbiol* **65**: 3527-3534.

Kip, N., Ouyang, W., van Winden, J., Raghoebarsing, A., van Niftrik, L., Pol, A. et al. (2011) Detection, isolation, and characterization of acidophilic methanotrophs from Sphagnum mosses. *Appl Environ Microbiol* **77**: 5643-5654.

Knapp, C.W., Fowle, D.A., Kulczycki, E., Roberts, J.A., and Graham, D.W. (2007) Methane monooxygenase gene expression mediated by methanobactin in the presence of mineral copper sources. *Proc Natl Acad Sci U S A* **104**: 12040-12045.

Lindner, A.S., Pacheco, A., Aldrich, H.C., Costello Staniec, A., Uz, I., and Hodson, D.J. (2007) Methylocystis hirsuta sp. nov., a novel methanotroph isolated from a groundwater aquifer. *Int J Syst Evol Microbiol* **57**: 1891-1900.

Muyzer, G., De Waal, E.C., and Uitterlinden, A.G. (1993) Profiling of complex microbial populations by denaturing gradient gel electrophoresis analysis of polymerase chain reaction-amplified genes coding for 16S rRNA. *Appl Environ Microbiol* **59**: 695-700.

Nguyen, A.D., Park, J.Y., Hwang, I.Y., Hamilton, R., Kalyuzhnaya, M.G., Kim, D., and Lee, E.Y. (2020) Genome-scale evaluation of core one-carbon metabolism in gammaproteobacterial methanotrophs grown on methane and methanol. *Metabolic engineering* **57**: 1-12.

Prior, S.D., and Dalton, H. (1985) The effect of copper ions on membrane content and methane monooxygenase activity in methanol-grown cells of Methylococcus capsulatus (Bath). *Microbiology* **131**: 155-163.

Semrau, J.D., Jagadevan, S., DiSpirito, A.A., Khalifa, A., Scanlan, J., Bergman, B.H. et al. (2013) Methanobactin and MmoD work in concert to act as the ‘copper‐switch’ in methanotrophs. *Environ Microbiol* **15**: 3077-3086.

Suleimanov, R., Tikhonova, E., Oshkin, I., Danilova, O., and Dedysh, S. (2023) Methylomonas montana sp. nov., the first nonpigmented methanotroph of the genus Methylomonas, isolated from mountain river sediments. *Microbiology* **92**: 766-774.

Takeuchi, M., Kamagata, Y., Oshima, K., Hanada, S., Tamaki, H., Marumo, K. et al. (2014) Methylocaldum marinum sp. nov., a thermotolerant, methane-oxidizing bacterium isolated from marine sediments, and emended description of the genus Methylocaldum. *Int J Syst Evol Microbiol* **64**: 3240-3246.

Tays, C., Guarnieri, M.T., Sauvageau, D., and Stein, L.Y. (2018) Combined effects of carbon and nitrogen source to optimize growth of proteobacterial methanotrophs. *Front Microbiol* **9**: 2239.

Tikhonova, E.N., Grouzdev, D.S., Avtukh, A.N., and Kravchenko, I.K. (2021) Methylocystis silviterrae sp. nov., a high-affinity methanotrophic bacterium isolated from the boreal forest soil. *Int J Syst Evol Microbiol* **71**: 005166.

Vorobev, A.V., Baani, M., Doronina, N.V., Brady, A.L., Liesack, W., Dunfield, P.F., and Dedysh, S.N. (2011) Methyloferula stellata gen. nov., sp. nov., an acidophilic, obligately methanotrophic bacterium that possesses only a soluble methane monooxygenase. *Int J Syst Evol Microbiol* **61**: 2456-2463.

Wartiainen, I., Hestnes, A.G., McDonald, I.R., and Svenning, M.M. (2006a) Methylocystis rosea sp. nov., a novel methanotrophic bacterium from Arctic wetland soil, Svalbard, Norway (78 N). *Int J Syst Evol Microbiol* **56**: 541-547.

Wartiainen, I., Hestnes, A.G., McDonald, I.R., and Svenning, M.M. (2006b) Methylobacter tundripaludum sp. nov., a methane-oxidizing bacterium from Arctic wetland soil on the Svalbard islands, Norway (78 N). *Int J Syst Evol Microbiol* **56**: 109-113.

Whittenbury, R., Phillips, K., and Wilkinson, J. (1970) Enrichment, isolation and some properties of methane-utilizing bacteria. *Microbiology* **61**: 205-218.

Wise, M.G., McArthur, J.V., and Shimkets, L.J. (2001) Methylosarcina fibrata gen. nov., sp. nov. and Methylosarcina quisquiliarum sp. nov., novel type 1 methanotrophs. *Int J Syst Evol Microbiol* **51**: 611-621.
